# Expressional diversity of grapevine 3-Hydroxy-3-methylglutaryl-CoA reductase (*VvHMGR*) in different grapes genotypes

**DOI:** 10.1186/s12870-021-03073-8

**Published:** 2021-06-19

**Authors:** Ting Zheng, Lubin Guan, Kun Yu, Muhammad Salman Haider, Maazullah Nasim, Zhongjie Liu, Teng Li, Kekun Zhang, Songtao Jiu, Haifeng Jia, Jinggui Fang

**Affiliations:** 1 College of Horticulture, Nanjing Agricultural University, Jiangsu Province, Nanjing City, 210095 PR China; 2 College of Agriculture, Shihezi University, Shihezi City, 832003 PR China; 3 College of Enology, Northwest A&F University, Yangling, 712100 PR China; 4 Department of Plant Science, Shanghai Jiao Tong University, 200030 Shanghai, PR China

**Keywords:** Grape, HMGR, Terpenoids, Color, Aroma

## Abstract

**Background:**

3-Hydroxy-3-methylglutaryl-CoA reductase (HMGR) is a key enzyme in the mevalonate (MVA) pathway, which regulates the metabolism of terpenoids in the cytoplasm and determines the type and content of downstream terpenoid metabolites.

**Results:**

Results showed that grapevine HMGR family has three members, such as *VvHMGR1*, *VvHMGR2*, and *VvHMGR3*. The expression of *VvHMGRs* in 'Kyoho' has tissue specificity, for example, *VvHMGR1* keeps a higher expression, *VvHMGR2* is the lowest, and *VvHMGR3* gradually decreases as the fruit development. *VvHMGR3* is closely related to *CsHMGR1* and *GmHMGR9* and has collinearity with *CsHMGR2* and *GmHMGR4*. By the prediction of interaction protein, it can interact with HMG-CoA synthase, MVA kinase, FPP/GGPP synthase, diphosphate mevalonate decarboxylase, and participates in the synthesis and metabolism of terpenoids. *VvHMGR3* have similar trends in expression with some of the genes of carotenoid biosynthesis and MEP pathways. *VvHMGR3* responds to various environmental and phytohormone stimuli, especially salt stress and ultraviolet (UV) treatment. The expression level of *VvHMGRs* is diverse in grapes of different colors and aroma. *VvHMGRs* are significantly higher in yellow varieties than that in red varieties, whereas rose-scented varieties showed significantly higher expression than that of strawberry aroma. The expression level is highest in yellow rose-scented varieties, and the lowest in red strawberry scent varieties, especially ‘Summer Black’ and ‘Fujiminori’.

**Conclusion:**

This study confirms the important role of *VvHMGR3* in the process of grape fruit coloring and aroma formation, and provided a new idea to explain the loss of grape aroma and poor coloring during production. There may be an additive effect between color and aroma in the HMGR expression aspect.

**Supplementary Information:**

The online version contains supplementary material available at 10.1186/s12870-021-03073-8.

## Background

Terpenoids are natural substances widely found in nature, composed of different numbers of isoprene units, and play an important role in the growth and development of plants. Firstly, terpenoids are the synthetic precursors of key active ingredients in plants, affecting the synthesis of brassinosteroid (BR), abscisic acid (ABA), carotenoids, etc. [1, 2]. Secondly, they help regulate plant organ development and energy metabolism, closely related to bud dormancy, organ shedding, biofilm system construction, and embryo, seed, and flower development. It also plays an important role in plant photosynthesis and respiration [3–6]. For example, the biosynthesis of phytosterols is essential for membrane fluidity and plant growth and development [7]. The six major hormones play important roles in plants, among these, ABA, gibberellins (GA), and cytokinins (CK) are isoprenoid compounds, and BR has sterol-based structures [8]. Thirdly, it is closely related to the formation of fruit quality. Terpenoids are a key component of the unique aroma of fruit. The terpenoid metabolic pathway is the most important pathway for the synthesis of special aroma substances in rose fragrance grapes [9]. Carotenoids, the product of the MEP metabolic pathway, contain multiple conjugated double bonds, which can form lycopene, capsicum red pigment, and other substances after cyclization to reveal the wide array of colors [10]. Studying the synthesis of terpenoids and their regulation mechanism is an urgent need to increase the content of terpenoids and achieve the improvement of fruit quality.

In the plant cells, two distinct pathways are responsible for the biosynthesis of terpene compounds, the cytosolic mevalonate pathway (MVA pathway) and the plastidial 2-C-methyl-D-erythritol-4-phosphate pathway (MEP pathway) [11, 12]. HMG-CoA reductase (HMGR, EC:1.1.1.34) is the first rate-limiting enzyme of the MVA pathway, which can catalyze the formation of MVA from HMG-CoA. It is an important regulatory site of the MVA pathway and plays a key role in the synthesis of cytoplasmic terpenoids [13, 14]. HMGR was discovered in 1958 and purified in 1986[15, 16]. It was first cloned in *Arabidopsis* in 1989 [17]. HMGR can catalyze the rate-limiting reaction in cholesterol biosynthesis, and it is one of the most regulated enzymes, so it has been deeply studied in animals and yeast [18]. Compared with animals, terpenoids play more roles in plants, and the regulation of HMGR activity in plants is more diverse. It not only plays a key regulatory role in the normal growth and development of plants but also necessary for plants to adapt to different environmental conditions [19–21].

Until now, HMGR genes have been isolated and cloned from more than 80 plants such as potato, Arabidopsis, rice, pear, tomato, etc., [14, 22–25]. However, little focus has been paid to the key enzyme of the terpene synthesis pathway in the grape genome, and the regulatory mechanism of *HMGR* genes during grape fruit development [9]. Therefore, this study aims to identify and analyze the HMGR family in *Vitis*, in-depth study of the structural characteristics and evolutionary relationship of VvHMGRs, analyze the tissue-specific expression pattern of *VvHMGRs*, and compare the changes of *VvHMGRs* in different colors and aroma types during fruit development stages, and analyze the response pattern of *VvHMGRs* to hormone and osmotic stress.

## Materials and methods

### Plant Materials and Treatment

Five-year-old grape varieties ‘Wink’ ‘Fujiminori’ ‘Kyoho’ ‘Summer Black’ ‘Red Globe’ ‘Jing Zaojing’ ‘Centennial Seedless’ ‘Yellow Italia’ ‘Red Italia’ and ‘Shine-Muscat’ were selected as the test material, grown in the Baima Experimental Vineyard, Nanjing Agricultural University (31°36′N, 119°10′E), Nanjing, China. The sampling was conducted on school land, and we confirm that the land owner gave permission for this and had undertook the formal identification of the samples. The grapevines were under routine management. At different stages of fruit development, we collected grape berries, peeled off grape skins, at 7, 8, 9, 10, 11, 12, 13, 14 weeks after flowering (7WAF, 8WAF, 9WAF, 10WAF, 11WAF, 12WAF, 13WAF, 14WAF). Potted 2-years-old grape varieties ‘Kyoho’ was also grown in the experimental field. We collected the different parts of grapes including young root, stem, leave, tendril, flower, flesh, skin, seed and xylem sap. Xylem sap was collected according to the instructions described by Zheng et al. [26, 27]. The samples were collected and immediately frozen in liquid nitrogen individually. All samples were stored at –80 °C for subsequent analysis.

### Bioinformatics analysis

We used HMMER 3.0 and Pfam protein family databases (http://pfam.xfam.org) and found domains including PF00368. Domains were used to search HMGR genes in the Grape Genome Database (CRIBI. http://genomes.cribi.unipd.it/grape/, Version 2.1.). The predicted genes were further confirmed for the existence of the conserved domains using SMART (http://smart.embl-heidelberg.de/smart/set_mode.cgi?GENOMIC=1) and INTERPROSCAN (http://www.ebi.ac.uk/interpro/search/sequence-search) programs. HMGR sequences of *Arabidopsis thaliana*, *Citrus sinensis*, *Glycine max*, *Populus trichocarpa,* and *Solanum lycopersicum* were downloaded from NCBI [28].

The number of amino acids, theoretical Mw, theoretical pI, aliphatic index, and grand average of hydropathicity of protein were analyzed by Ex-PaSy (http://web.expasy.org/protparam/). TMPred (http://www.ch.embnet.org/software/TMPRED_form.html) and TMHMM (http://www.cbs.dtu.dk/services/TMHMM/) were used to further analyzed transmembrane domain, GOR4 (https://npsa-prabi.ibcp.fr/cgi-bin/npsa_automat.pl?page=npsa_gor4.html) to analyzed secondary structure, and SWISS-MODEL (https://swissmodel.expasy.org/) to predict tertiary structure. Chromosomal distribution was determined by Maplnspect software according to the CRIBI database. Phylogenetic trees were constructed using the Maximum Likelihood (ML) method with MEGA 6 (Sudhir Kumar, Arizona State University, USA). The reliability of the obtained trees was tested using bootstrap with 1000 replicates. Gene synteny was analyzed with TBtools software (Version 066) using the MCScanX with gene duplication parameters [29].

Gene structure and motifs were determined the exon and intron regions by Gene Structure Display Server (http://gsds.cbi.pku.edu.cn/) and found the coding sequence (CDS) and the correspondent full-length gene sequences in NCBI. The conserved motifs were constructed in the MEME program (http://meme-suite.org/tools/meme) using full-length amino acid sequences, as the default setting was 20 for the motif number. We considered the 1500 bp upstream of *VvHMGRs* as promoter regions and performed cis-regulatory element analysis in PlantCARE (http://bioinformatics.psb.ugent.be/webtools/plantcare/html/). The interaction protein of VvHMGRs was analyzed and predicted using the online software STRING (https://string-db.org).

### HMGR enzyme activity determination

The extraction of HMGR in the fruit during development stages in 'Kyoho' grape was carried out according to the method described by Kim et al. [14] and the enzyme activity was detected using an enzyme-linked immunosorbent assay (ELISA) kit (Lvye Biotechnology Co. Ltd., Yancheng, Jiangsu, China).

### Aroma components determination

Berry skin and berry flesh of 10 varieties at the mature stage was separated to measure the aroma components. 3 g material after grinding is dissolved in 3 mL NaCl for determination. Samples were analyzed with a gas chromatograph (TRACE 1310, Thermo Scientific), coupled to a triple quadrupole mass spectrometer (TSQ 9000, Thermo Scientific). One microliter of each sample was injected in split mode (ratio 1: 5) with about 17% of injected samples being transported by a carrier gas into a non-polar column (TG-5MS, 30 m, 0.25 mm ID, 0.25 μm film thickness, Thermo Scientific). Compounds were tentatively identified by mass spectrometry analyses: i.e., matching mass spectrum of samples with database in NIST mass spectral library. 3-octanol was used as an internal standard substance [9, 30].

### Hormone and osmotic stress treatment of grape fruits

We prepared the different concentrations of ethylene (ETH, 200, 500, and 2000 mg·L^−1^), methyl jasmonate (MeJA, 5, 50, and 100 μmol·L^−1^), Salicylic acid (SA, 100, 250, 500 μmol·L^−1^), auxin (IAA, 0.1, 10, 20 μmol·L^−1^), and NaCl solution (20, 60, and 100 mM), polyethylene glycol (PEG, -0.2, -0.4, -0.6 MPa), NaOH (pH 5, 7, 9). According to the method of Zheng et al. [31], the grape fruits of ‘Kyoho’ for hormone and osmotic stress were immersed in the solution and vacuumed three times, each time for 10 min. Because ethylene is in a gaseous state, we use ethephon to simulate the effect of ethylene and place it in a sealed box after treatment. Distilled water was used as a control. Each treatment was repeated three times with 10 fruits in each group. Sampling was stored in an airtight box and performed at 1, 12, 24, and 48 h after hormone and osmotic stress exposure, at 24, 36, 48, 60 h after light and temperature stress. qRT-PCR was performed to detect the relative gene expression and calculated using the 2^–△Ct^ method so that all treatments can be compared at the same level.

The Heml 1.0.3.7 software was used to study the expression of *VvHMGRs* in different grapes during the different developmental stages of grapevines.

### Light and temperature stress treatment of grape fruits

The grape fruit were placed in a different incubator with various temperature (4, 25, 37, and -20℃), the room temperature (27℃) was used as a control, and treated with 254 nm Ultraviolet-C radiation (UV-C) for 60, 90, 120 min, respectively, white light was used as control. Sampling was performed at 24, 36, 48, 60 h after light and temperature stress.

### RNA extraction, cDNA synthesis, and qRT-PCR

Total RNA was extracted using the cetyltrimethylammonium bromide (CTAB) method by FastPure Plant Total RNA Isolation Kit (Vazyme, China). The RNA purity and integrity were assessed based on the A260/A280 absorbance ratio and 1.0% agarose gel electrophoresis. cDNA was synthesized using a HifairII® 1st Strand cDNA Synthesis SuperMix for qPCR (Yeasen, Shanghai, China).

The qRT-PCR comprised 5 μL SYBR Premix Ex Taq™ (Yeasen, Shanghai, China), 0.3 μL of each primer (10 μM), 2 μL cDNA, and 2.4 μL RNase-free water in a total volume of 10 μL. The reaction was performed using a LightCycler 1.5 instrument (Roche, Germany), with the preliminary step at 95 °C for 30 s followed by 35 cycles at 95 °C for 5 s and 58 °C for 35 s. The expression of genes related to coloration and aroma formation was calculated using the 2^–△Ct^ method. Oligo d(T) primers were used for cDNA synthesis and gene specific primers are listed in Table S1.

### Statistical analysis

All data (at least three replications, *N* = 3) were presented as means with standard errors (SEMs). The mean ± SEM values were calculated for each treatment using Microsoft Excel (Microsoft Corporation, Albuquerque, NM, USA). Statistical analysis of variance (ANOVA) was performed using SPSS 17.0 (SPSS, Inc., Chicago, IL, USA) with Duncan's multiple range test at P < 0.05. Heml 1.0.3.7 and Origin Pro 9 (Origin Inc., Northampton, MA, USA) was used to produce the figures.

## Results

### Identification and evolutionary analysis of *VvHMGRs* genes in grape

**Fig.1:**
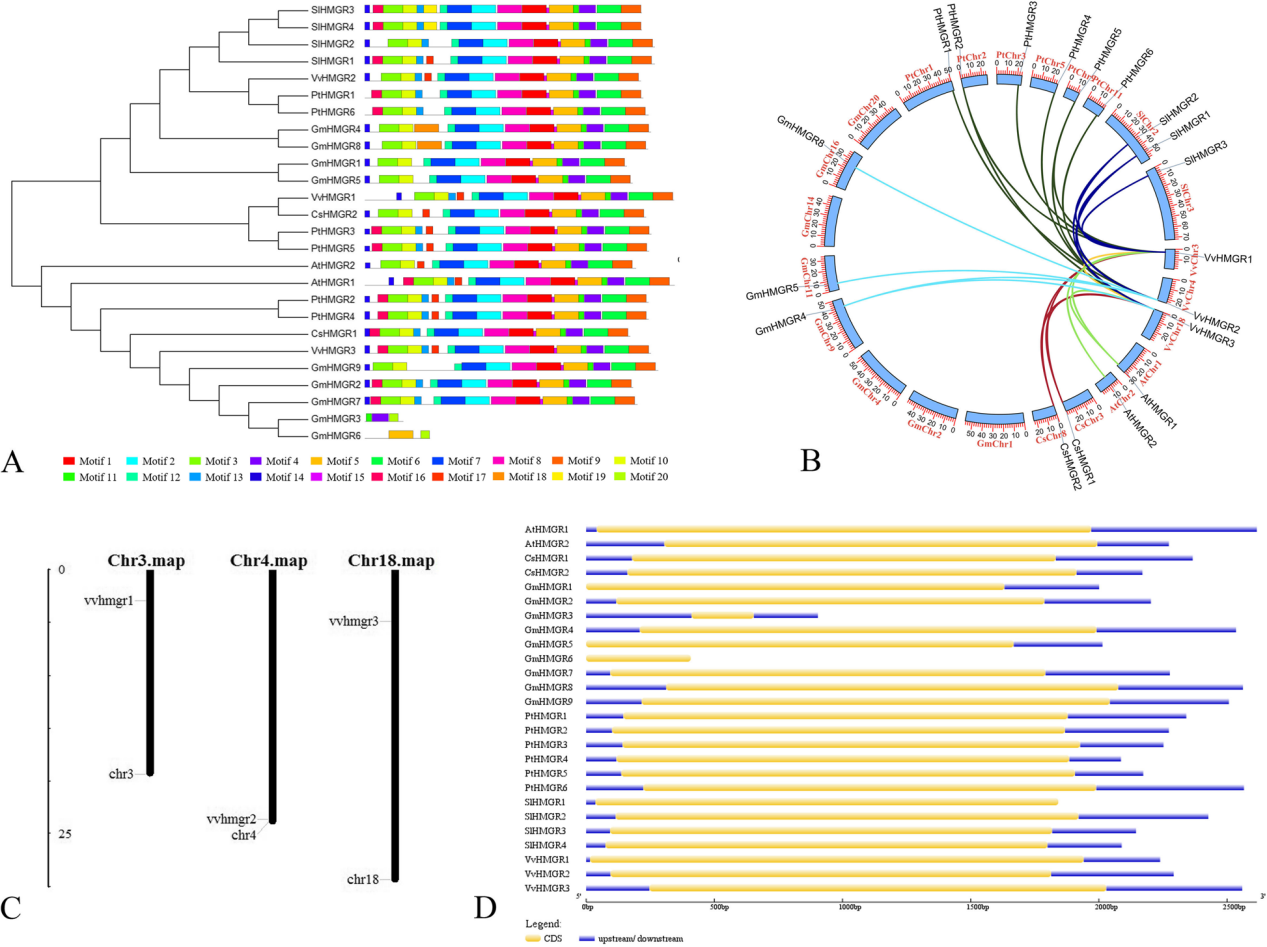
Evolution and structure of *VvHMGRs*. **A** The evolutionary tree and protein structure of seven species of HMGR. **B** Collinearity analysis of seven species. **C** Chromosome location of *VvHMGRs*. **D** Gene structure of seven species of HMGR

We predicted *VvHMGR* in the genome sequence scaffolds of Pinot noir grape and used HMMER 3.0 and Pfam protein family databases to search sequence homologs in grape genomes. SMAT and INTERPROSCAN programs were used to verify the predicted genes, which were three *VvHMGRs* in grape, *VvHMGR1* (XM_002283147), *VvHMGR2* (XM_002265602), *VvHMGR3* (XM_002275791). Three *VvHMGRs* are located on chromosomes 3, 4, and 18, respectively (Fig. 1C), and the length of the coding region is between 1710 and 1930 bp, which is the same as *Arabidopsis thaliana, Citrus sinensis, Glycine max, Populus trichocarpa,* and *Solanum lycopersicum*. The information of *HMGRs* genes of 7 species is listed in Table S2. The secondary structure of all *HMGRs* genes is dominated by α helix and random coils, accounting for 80% (Table S3), and all are located at the endoplasmic reticulum (Table S4).

According to the analysis of the evolutionary relationship (Fig. 1A), *VvHMGR1* is closely related to *CsHMGR2, PtHMGR3* and *PtHMGR5*, and *VvHMGR2* closest to *PtHMGR1* and *PtHMGR6*, while *VvHMGR3* closest to *CsHMGR1* and *GmHMGR9*, which has collinearity with *CsHMGR2* and *GmHMGR4* (Fig. 1B). HMGR is highly conserved among different species (Fig. 1A, Fig. S1). It contains 14 common motifs (Fig. S2). The structures of VvHMGR1 and VvHMGR2 are consistent. Compared with other members, VvHMGR3 has motif16. All *HMGR* genes from selected species contain no introns, and at least one UTR region except for *GmHMGR6* (Fig. 1D). The gene structure of *VvHMGRs* is consistent, with the same number and different lengths of UTRs.

### Tissue expression of HMGR family in ‘Kyoho’ grape

**Fig. 2:**
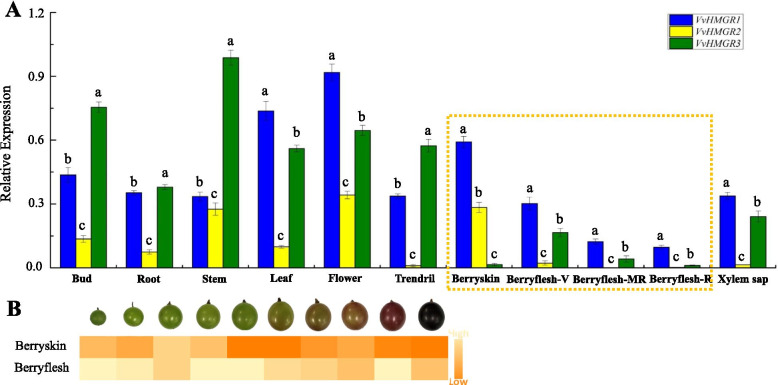
Gene expression levels of *VvHMGRs* in various tissues and enzyme activities in the fruit during development stages in 'Kyoho' grape. **A** *VvHMGRs* genes expression in different grape tissues. **B** HMGR activities during fruit development. Berry Flesh-V indicates berry flesh in véraison period; Berry Flesh-MR indicates berry flesh in mid-ripening period; Berry Flesh-R indicates berry flesh in ripening period. Vertical bars represented standard deviations (SD) of means (*n* = 3). Different letters indicated a statistical difference at *P* < 0.05 as determined by Duncan’s multiple range test

We detected the expression levels of 3 HMGRs family members in the bud, root, stem, leaf, flower, tendril, exocarp, pulp, and xylem sap of 'Kyoho' grape. The results showed that the expression of *VvHMGRs* in 'Kyoho' had obvious organization specificity. As shown in Fig. 2A, *VvHMGR1* has always been highly expressed, and its expression levels in leaves, flowers, and exocarp was much higher than in other tissues. The expression level of *VvHMGR2* was the lowest among the three members, and expression level in stems, flowers and exocarp were higher than in other tissues. The expression level of *VvHMGR3* was low in peel and pulp and higher in other tissues. As the fruit develops, the expression level of *VvHMGR3* was getting lower gradually. By measuring the enzyme activity of HMGR during the fruit development process, as a whole, the enzyme activity in peel and pulp showed a decreasing trend, and higher in pulp than peel (Fig. 2B). This phenomenon indicates that *VvHMGR3* is more directly related to the enzymatic activity of HMGR, and its expression greatly affects the enzymatic activity. Therefore, the enzyme activity in the peel is lower than that in pulp.

### Expression patterns of *VvHMGRs* during different developmental stages in different types of grapes

**Fig. 3:**
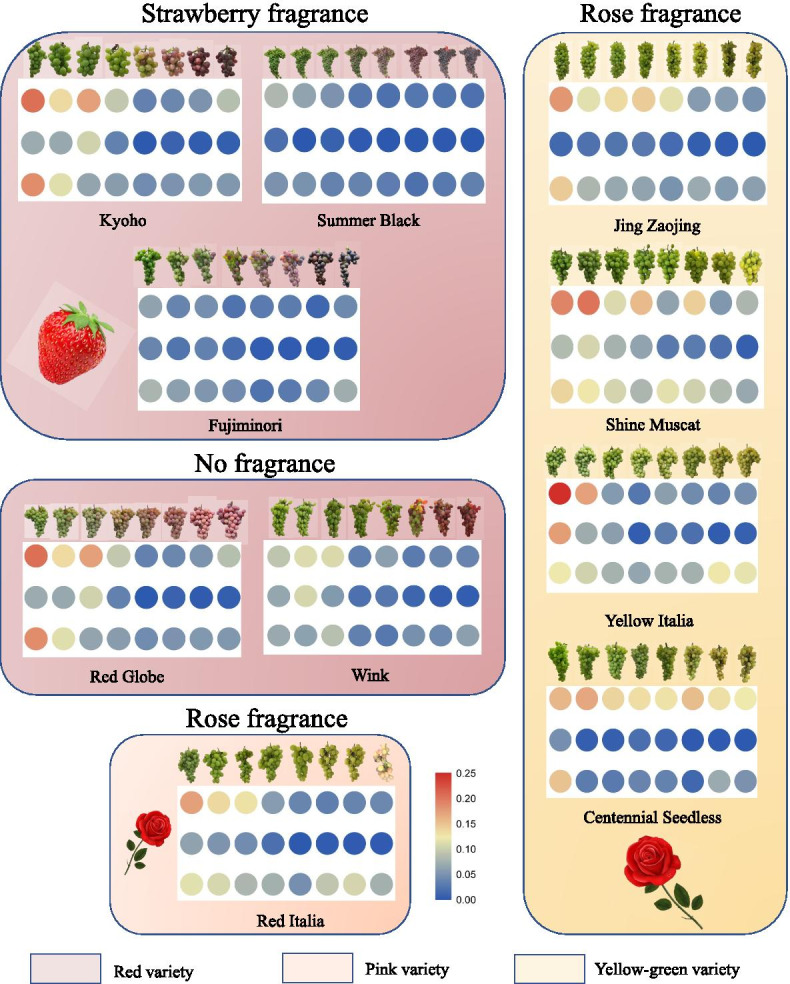
The expression levels of *VvHMGRs* during fruit development in 10 different types of cultivars. Each group represent *VvHMGR1, VvHMGR2, VvHMGR3* from top to bottom. Every column represents 7WAF, 8WAF, 9WAF, 10WAF, 11WAF, 12WAF, 13WAF, 14WAF from left to right

Through the detection of expression levels of *VvHMGRs* in ten grape varieties, we found that *VvHMGR1* has always maintained a relatively high expression level during the fruit development of all varieties, especially in yellow rose fragrance varieties (Fig. 3). The expression level of *VvHMGR2* was always low, not affected by the fruit development stage in ‘Summer Black’ ‘Fujiminori’ ‘Jing Zaojing’ and ‘Centennial seedless’, while decreased gradually in other varieties. The expression level of *VvHMGR3* in rose fragrance varieties (‘Shine Muscat’ and ‘Yellow Italia’) was higher than that of other varieties, and it showed a trend of first decreasing and then increasing during fruit development, which was related to the enlargement of early fruit and strong terpenoid metabolism as aroma formation in the later fruit. However, this phenomenon did not exist in strawberry fragrance fruits.

Overall, the expression levels of *VvHMGRs* in different color fruits were different, and the expression level in red fruits was low, especially in ‘Summer Black’ and ‘Fujiminori’. In the yellow varieties with rose fragrance, the expression level of *VvHMGRs* was the highest, and in the red varieties with strawberry fragrance was the lowest. The expression level of *VvHMGR3* in yellow fruit was overall high, especially deep yellow color variety ‘Shine Muscat’ and ‘Yellow Italia’, while ‘Jingzaojing’ and ‘Centennial seedless’, which are mainly green, showed relatively low expression. In the sampling process, we found that the yellow color of ‘Red Italian’ was deepening in the early stage of fruit development, and it was pale pink when mature. The expression pattern of *VvHMGR3* was similar to the yellow variety, which also confirmed this phenomenon.

### Aroma components in different types of grapes

**Fig. 4:**
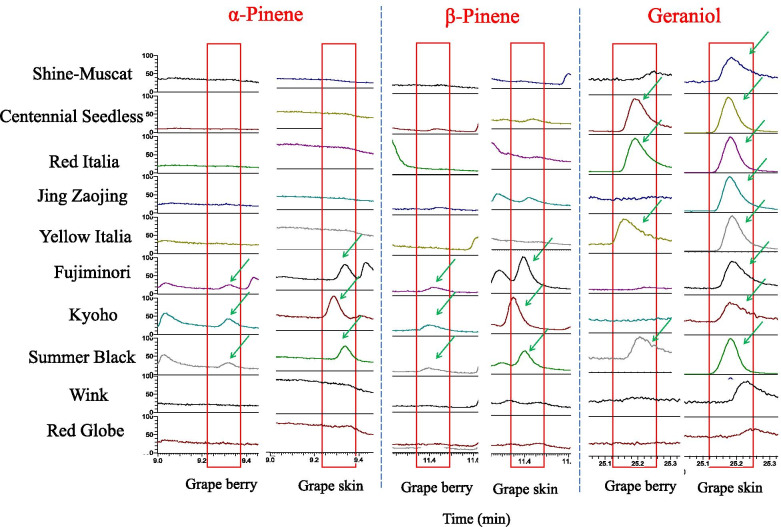
Characteristic aroma components of 10 varieties in berry skin and berry flesh

The aroma components of 10 varieties in berry skin and berry flesh were measured at the mature stage, and results showed that the aroma components in the berry skin were more abundant than that in berry flesh (Fig. S3, Table S5). There were 18, 20, 20, 26, 33 types of aroma in berry skin more than that in berry flesh in rose-scented varieties, 29, 37, 39 types in strawberry-scented varieties, and 20, 26 in non-fragrant varieties. The main terpene aromas in grapes included linalool and geraniol, of which geraniol was mainly in the rose-scented varieties. We found that β-pinene and α-pinene were mainly volatile aroma components in strawberry-scented varieties (Fig. 4). Linalool is the key component of terpene aroma in grapes, found in different scented varieties and occupied a large proportion. ‘Red Italia’ was a red rose-scented variety with many unique ingredients, different from red strawberry-scented varieties, red non-fragrant and green rose-scented varieties. D-limonene was rich in berry skin and berry flesh of ‘Red Italia’, cis-linaloloxide and trans-rose oxide were also specifically detected in ‘Red Italia’. In addition, we also detected germacrene D,which belong to the sesquiterpene aroma in ‘Jing Zaojing’ and ‘Summer Black’.

### Changes of terpenoids and anthocyanin metabolism associated genes expression and their correlation with *VvHMGRs*

**Fig. 5:**
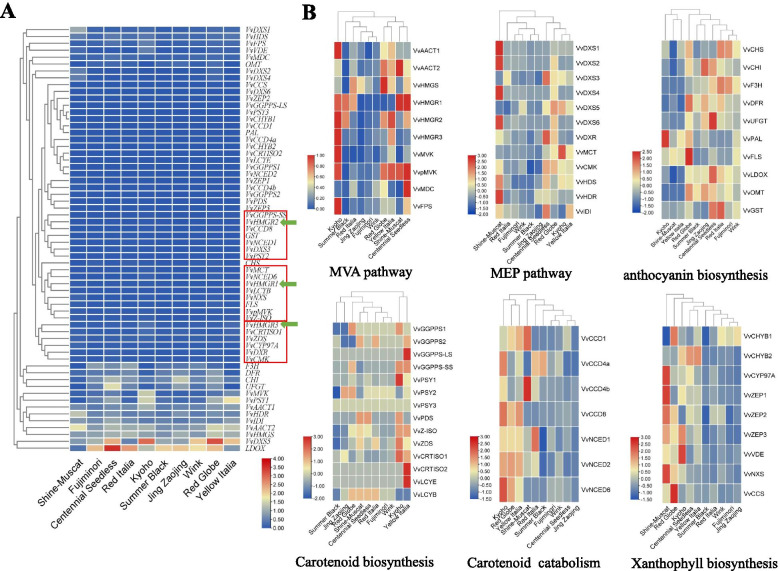
The expression of genes related to aroma formation and coloration in skins from ten varieties at the mature stage. **A** All gene expression levels, cluster rows in the left. **B** The gene expression levels of six pathways after row scale and cluster cols, including MVA pathway, MEP pathway, anthocyanin biosynthesis, carotenoid biosynthesis, carotenoid catabolism, and xanthophyll biosynthesis

The gene expression levels of aroma formation and color-related pathways were tested in skins from ten grape varieties at the mature stage. After gene-level clustering (Fig. 5A), we found that *VvHMGR3* had similar trends in expression with *VvCRTISO1, VvZDS, VvCYP97A, VvDXR,* and *VvCMK*. *VvHMGR1* was similar with the changes of *VvMCT, VvNCED6,* and *VvLCYB*. *VvHMGR2* was similar with *VvGGPPS-SS* and *VvCCD8*, which means that *VvHMGRs* might be linked to the carotenoid biosynthesis and MEP pathways.

Because of the high expressed genes concealing the changes of other genes, we have carried out the normalization of genes to show each gene to the greatest extent. Through sample-level clustering (Fig. 5B), the MVA pathway genes expression patterns of typical yellow rose-scented varieties ‘Shine-Muscat’ were the same with ‘Centennial Seedless’, and clustered together with ‘Yellow Italia’. The MEP pathway gene expression pattern of ‘Shine-Muscat’ were different from others, and its anthocyanin biosynthesis pathway was consistent with the changes in ‘Yellow Italia’, carotenoid biosynthesis was similar to ‘Centennial Seedless’, but carotenoid catabolism and xanthophyll biosynthesis were different from others. The typical red cultivars ‘Wink’ and ‘Fujiminori’ were clustered together in all six pathways, suggesting that these two cultivars were very similar in terms of terpenoid synthesis and metabolism and anthocyanin biosynthesis.

### HMGR plays an important role in grape growth and development

By analyzing the transmembrane structure, VvHMGRs protein exhibited transmembrane properties. VvHMGR1 had 9 inside to outside (i-o) helices, 9 outside to inside helices (o-i), VvHMGR2 had 7 i-o and 8 o-i, and VvHMGR3 had 6 i-o and 5 o-i (Fig. S4A). The secondary structures of VvHMGRs were quite different, composed of alpha-helix, extended strand, and random coil, and distributed throughout the protein (Fig. S4B). The tertiary structure was highly conserved, and its catalytic region contains L, N, and S three binding domains. The L domain is the largest, with two HMG-CoA binding domains and one NADP(H) binding domain, while the small spiral S domain contains another NADP(H) binding domain (Fig. S4C).

The computational predictions for cis elements in the promoter of the *VvHMGRs* genes suggest that they are in a complex network of environmental and phytohormone regulation (Fig. S4D). Through the interaction protein prediction based on protein structure, VvHMGRs possibly interact with HMG-CoA synthase, MVA kinase, FPP/GGPP synthase, and diphosphate mevalonate decarboxylase confirming that the most important effect of HMGR was to take part in the synthesize and metabolize of terpenoids based on MVA pathways (Fig. S4E).

Combined with evolutionary bioinformatics analysis (Fig. 1, S4), tissue expression pattern (Fig. 2), and expression pattern of *VvHMGRs* in different types of fruits (Fig. 3, 5), we predicted *VvHMGR3* play an important role in the growth and development process of grape.

### VvHMGR3 responds to external conditions

**Fig. 6:**
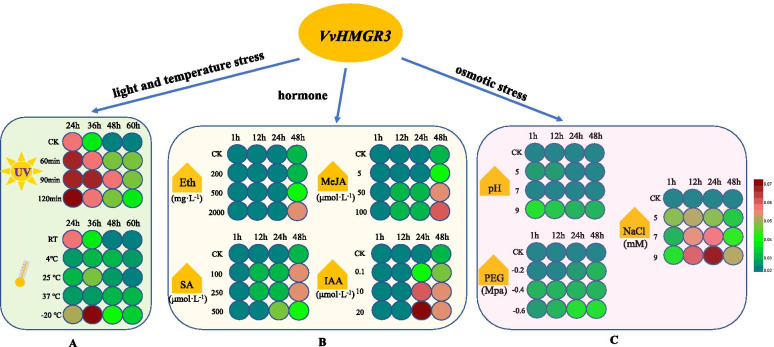
Response mode of *VvHMGR3* to external conditions by qRT-PCR detection. **A** Light and temperature stress, including UV and temperature treatment; **B** Hormone treatment, including Eth, MeJA, SA, IAA. **C** Osmotic stress, including pH, PEG, NaCl

We can see the variation of *VvHMGR3* under different pH, osmotic stress, NaCl concentration, temperature, and UV treatment time from Fig. 6. On the whole, *VvHMGR3* was regulated by salt stress, temperature stress, and ultraviolet radiation, and it also had different responses to four hormones. The final expression of *VvHMGR3* was up-regulated under Eth, MeJA, SA and IAA treatment, and the response time was different, Eth was 48 h, AA was 24 h, MeJA and SA were 12 h, respectively. For the concentration of treatment, *VvHMGR3* was affected by the high concentration of MeJA and medium concentration of SA, the expression of VvHMGR3 was proportional to the hormone concentration except for SA.

In osmotic stress treatment, the response of *VvHMGR3* to pH and PEG was lower than that of NaCl, which was manifested as a relatively alkaline environment, high osmotic value, and high salt content. The expression of *VvHMGR3*increased sharply at 24 h and then decreased slowly under normal conditions, while under UV treatment, the expression of *VvHMGR3* was much higher than normal grapes at 24 h, indicating that UV promoted the expression of *VvHMGR3*. During the postharvest storage, ultra-low temperature treatment (-20 °C) slowed down the high expression of *VvHMGR3*, while high temperature (25 °C and 37 °C) and ordinary low temperature (4 °C) decreased the expression of *VvHMGR3*. In summary, *VvHMGR3* was regulated by hormone, osmotic stress, light and temperature stress, especially salt stress, and UV treatment.

## Discussion

### *VvHMGRs* are structurally conserved and functionally diverse

HMGR protein is composed of three parts, transmembrane domain (N-terminal), catalytic domain (C-terminal), and connecting domain [32, 33]. Transmembrane domains are highly variable in evolution, and the negative regulation of HMGR activity in plants depends more on the N-terminal domain [19, 33]. The catalytic domain is relatively conserved and consists of the N domain, L domain, and S domain. The L domain contains two HMG-CoA binding sites and one NADP (H) binding site, and the S domain contains another NADP (H) binding site [33]. Plant HMGR is under the control of complex mechanisms operating at both transcriptional and post-transcriptional levels. Due to the presence of conserved serine sites in the catalytic domain, the activity change of HMGR is a process regulated by phosphorylation. The phosphorylation state of AtHMGR1S at Ser577 is very important for regulating HMGR activity in Arabidopsis [34]. The secondary structure of the *HMGR* gene is dominated by α helix and random coil [35], which is also validated in this study.

HMGR exists as a small gene family that contains 2 members in Arabidopsis [36], 3 in potato [37], 5 in rubber tree [38, 39], 3 in rice [23], 4 in wheat [40], 4 in Salvia [41], 9 in cotton. The gene structure and protein architecture of all plant HMGR genes are highly conserved, derived from one ancestor gene, and finally developed into four distinct groups [28]. The function and evolution of the HMGR gene family is dramatically conserved throughout the plant kingdom [33, 42]. Through the analysis of the evolutionary relationship between grape and other species, we found that *VvHMGR1* is closely related to *Citrus sinensis* and *Populus trichocarpa*, *VvHMGR2* is closest to *Populus trichocarpa* and *Solanum lycopersicum*, and *VvHMGR3* is closest to *Citrus sinensis* and *Glycine max*, which was consistent with Li et al. [28].

The expression of different members of the HMGR gene family showed significant differences [39]. Taking *Salvia miltiorrhiza* as an example, *SmHMGR1* and *SmHMGR4* were highly expressed in flowers, *SmHMGR2* was mainly expressed in leaves and stems, and *SmHMGR3* was highly expressed in other tissues except for flowers [41]. Different members of the HMGR gene family in a species have distinct expression patterns, regulatory characteristics and physiological functions, and jointly regulate MVA metabolic pathways to determine the type and content of downstream terpene metabolites [25, 41, 43]. *HMGR1* is involved in the synthesis of sterols, while *HMGR2* and *HMGR3* are involved in the accumulation of sesquiterpenes in potatoes [37]. In this study, there are three members of the *HMGR* gene family in the grape. *VvHMGR1* is always in a high expression state, and *VvHMGR2* had the lowest. The expression level of *VvHMGR3* decreased with fruit development, which may be related to the different functions of different HMGR family members.

### HMGR plays an important role in plant growth and development

HMGR affects plant growth and development [44, 45]. The MVA pathway has a regulatory effect on cell growth, and cell growth depends on MVA and its derivatives [19]. HMGR protein was accumulated rapidly in the cell division stage in the skin during the early stage of melon fruit development, with active cell division occur in the peel, which determines the size of melon fruit [46]. The deletion of the *Arabidopsis* gene *AtHMGR1* leads to plant shortening, premature aging, male sterility, and lower sterol content than wild-type plants [47]. Inhibiting the HMGR activity with lovastatin can block MVA synthesis and lead to insufficient IPP synthesis, which leads to significant inhibition of tomato fruit size and development. After adding HMGR to catalyze MVA, it returns to normal [3]. We found that the expression patterns of *VvHMGRs* are different during the development of different varieties of grapes, which may be related to the demand for terpenoids during development. In addition, HMGR can positively regulate the metabolism of terpenoids, thereby affecting hormone synthesis, to influence plant growth and development [48]. Suppressing the expression of *VvHMGR3* can increase the content of IAA, ABA and BR contents, and reduce the content of GA_3_ and ZR contents [9].

HMGR is positively correlated with terpenoid production [44, 49, 50]. In this study, we confirmed that the expression level of *VvHMGRs* in different tissues was quite different. It may interact with HMG-CoA synthase, MVA kinase, FPP/GGPP synthase, and diphosphate mevalonate decarboxylase in the terpenoid metabolism pathway and participate in the synthesis of terpenoids, which is consistent with Dai et al. [25] and Hedl et al. [51], who reported that the main function of HMGR in plants is to participate in the synthesis of terpenoids in the MVA pathway. Adjusting the relative expression of key enzyme genes such as HMGR by biological means can change the production of related terpenoids [14, 52–54]. Overexpression of the *HMGR* can increase the contents of sterols, rubber and resin, saponins, and artemisinin in tobacco, guayule, ginseng, and artemisia, respectively [3, 13, 14, 55]. The silence of *HMGR* in *Centella asiatica* affected the synthesis of triterpene saponins, and heterologous expression of *Arabidopsis HMGR1* gene in tomato can greatly increase plant sterol content [56].

### HMGR participates in the formation of fruit pigment and aroma

HMGR regulated color formation by affecting the MEP pathway and the synthesis of anthocyanins [48, 57, 58]. Previous researchers found that HMGR can encode functional proteins and accelerate the biosynthesis of carotenoids from *Withania somnifera* and *Azadirachta indica* [58, 59]. In this study, it was also found that *VvHMGRs* had similar trends in the expression with many genes of carotenoid biosynthesis and MEP pathways, and the expression level of *VvHMGRs* in yellow varieties was generally higher than that in red varieties. These results represent that *VvHMGRs* are closely related to fruit coloring, which may depend on their effect on the active terpenoid metabolic pathways in yellow varieties. Overexpressed *HMGR* gene can also increase the content of lycopene in *Artemisia annua* [57], which also confirms the positive role of *HMGR* in isoprenoid biosynthesis. So we believe that HMGR might be associated with the synthesis of carotenoids, but the mechanism needs to be further studied. Besides, the biosynthesis of sesquiterpenes and triterpenes had an antagonistic effect on the accumulation of anthocyanins. Down-regulation of the *HMGR* gene can positively induce the expression of a variety of hormones and affect the accumulation of anthocyanins [48]. Overexpression of *VvHMGR3* inhibited the coloring of strawberry fruit, and inhibited expression promoted fruit coloring when *VvHMGR3* was expressed in strawberry fruits, confirming the direct relationship between HMGR and anthocyanin synthesis. Maybe it was related to primary metabolism (sterols, carotenoids, etc.) and secondary products from the phenyl ammonia lyase pathway. *VvHMGR3* affect fruit ripening process in strawberry. After the inhibition, the contents of IAA, ABA, and BR increased [9]. Combined with the results in this study, we confirmed that HMGR plays an important role in grapefruit coloring.

HMGR is involved in the regulation of aroma formation. Terpenoids are the precursors for the synthesis of main aroma substances in many plants, and the expression of the *HMGR* gene can affect the aroma by affecting the synthesis of terpenoids [14]. More than 70 kinds of terpenes have been identified in grapes, mainly monoterpenes, diterpenes, and sesquiterpenes [60, 61]. MVA pathway is mainly used to synthesize sesquiterpene and triterpene aroma components, which is directly regulated by HMGR. The separation of MVA and MEP pathways is not completely independent. MVA pathway synthesis products can enter plastid to form monoterpenes and diterpenes [6, 18]. The expression of the *HMGR* gene had an effect on aroma composition, and overexpression of *VvHMGR3* improved the types of aroma components in strawberry fruits, among which the linalool content, α-terpineol, and β-pinene significantly increased [9]. Obvious differences in terpenoid components existed in rose-scented and strawberry-scented varieties. Rose-scented grapes had many types and rich content of terpene aroma components [62]. We found that geraniol was mainly found in rose-scented varieties, while β-pinene and α-pinene were mainly found in strawberry-scented varieties. Linalool, the main terpene aroma of grapes, also had great differences in different types of aroma varieties. It was found that yellow cultivars were more likely to carry rose fragrance, which may be related to carotenoid synthesis and rose fragrance formation all belong to the terpene metabolic pathway. And the expression pattern of *VvHMGR3* was consistent with the changes of these two traits, which also confirms represented that it might play an important role in color and aroma formation.

### HMGR transcription changes in response to various environmental and phytohormone stimuli

The enzyme activity of HMGR is highly regulated by many factors such as plant growth and development, light, temperature, humidity, ultraviolet light, pathogens, exogenous growth regulators, sterols, and mechanical damage [14, 20, 63]. In this study, we confirmed that *VvHMGR3* was affected by hormones, light temperature stress, and osmotic stress. Appropriate stress can increase the activity of HMGR and promote the synthesis of terpenoids [20]. This study shows that pH and PEG have little effect on *HMGR*, while salt stress can effectively promote the expression of *HMGR*. The higher activity level of HMGR is usually associated with the rapid growth of plants, and the activity is greatly reduced in mature tissues [9]. Light inhibits HMGR activity, while dark induces HMGR gene expression. The response of HMGR to light is low, and this process is mediated by light-responsive elements such as GATA and SORLIP motifs [52, 64]. However, in this study, ultraviolet light up-regulated the expression of *VvHMGR3*, which means that light and ultraviolet light have different regulatory effects onHMGR activity. Moreover, the *HMGR* gene is significantly induced by ETH, MeJA, SA, ABA, etc., [65, 66]. ABA can inhibit the activity of HMGR by 40% in pea, but Zeatin (ZT) and GA increase its activity [67]. This study confirmed that *VvHMGR3* was up-regulated under Eth, MeJA, SA, and IAA treatment, while the response time was different, and it was more susceptible to high concentrations of MeJA and medium concentration of SA. The expression of *VvHMGR3* is directly proportional to the concentration of Eth, MeJA, and IAA hormones. Our findings are consistent with the study of Pu et al. [63] and Ma et al. [41], reporting that *HMGR* gene expression was up-regulated after using SA and MeJA. However, whether the effect of plant hormones on the activity of HMGR is due to their role as a feedback regulation of terpenoids or as a growth regulator is still unclear.

## Conclusion

By analyzing the structure and evolutionary characteristics of the *HMGR* gene family in grapes, detecting the expression levels of *VvHMGRs* in different tissues and different types of grapes, we confirmed the important role of VvHMGR3 in the process of grape fruit coloration and aroma formation, the expression level of which was relatively high in yellow and rose fragrance grape varieties. *VvHMGR3* is linked with some genes of carotenoid biosynthesis and MEP pathway, which can interact with MVA kinase, and its expression is regulated by hormones, osmotic stress, and light and temperature stress. These results provide more theoretical support for the formation of grape fruit quality and the synthesis mechanism of terpenoids (Fig. S5).

## Supplementary Information

**Additional file 1: Fig. S1.** The amino acid sequence of HMGRs motifs in MEME analysis. **Fig. S2.** The multiple alignments of deduced amino acid sequences of HMGRs. **Fig. S3.** Aroma components of 10 varieties in berry skin and berry flesh. **Fig. S4.** Protein structure and function analysis of VvHMGRs. **Fig. S5.** Synthetic pathways of main terpenoids in grapes.

**Additional file 2: Table S1.** Primer used for qRT-PCR on the grape. **Table S2.** Information on HMGR genes. **Table S3.** Analysis of protein secondary structure. **Table S4.** Prediction of subcellular localization. **Table S5.** Aroma components of 10 varieties in berry skin and berry flesh.

## Data Availability

All data generated or analyzed during this study are included in this published article and in its supplementary information files. The materials are available upon request by contacting the corresponding author. The datasets generated during the current study are available in the GenBank repository, http://www.ncbi.nlm.nih.gov/Genbank and the accession numbers are as follows: *VvHMGR1* (XM002283147), *VvHMGR2* (XM002265602), *VvHMGR3* (XM002275791), *AtHMGR1* (NM106299), *AtHMGR2* (NM127292), *CsHMGR1* (XM006473798), *CsHMGR2* (XM006486498), *GmHMGR1* (XM003517069), *GmHMGR2* (XM003519426), *GmHMGR3* (XM014775260), *GmHMGR4* (XM003534178), *GmHMGR5* (XM003537651), *GmHMGR6* (XM006596524), *GmHMGR7* (XM003545508), *GmHMGR8* (XM003547838), *GmHMGR9* (XM006605513), *PtHMGR1* (XM002300508), *PtHMGR2* (XM002301862), *PtHMGR3* (XM006384809), *PtHMGR4* (XM024601892), *PtHMGR5* (XM024607975), PtHMGR6 (XM002316990), *SlHMGR1* (NM001309881), *SlHMGR2* (NM001309190), *SlHMGR3* (XM004234541), *SlHMGR4* (XM010319372)..

## Abbreviations

HMGR: 3-Hydroxy-3-methylglutaryl-CoA reductase
MVA: Mevalonate
MEP: 2-C-methyl-D-erythritol-4-phosphate
BR: Brassinosteroid
ABA: Abscisic acid
ETH: Ethylene
MeJA: Methyl jasmonate
SA: Salicylic acid
IAA: Auxin
UV-C: Ultraviolet-C radiation
PEG: Polyethylene glycol
