# Supplementary material for: Expressional diversity of grapevine 3-Hydroxy-3-methylglutaryl-CoA reductase (VvHMGR) in different grapes genotypes
Source: BMC Plant Biol. 2021 Jun 19;21:279. doi: 10.1186/s12870-021-03073-8 (PMC8214791; doi:10.1186/s12870-021-03073-8)
Supplement: Supplementary file 1 — Additional file 1: Fig. S1. The amino acid sequence of HMGRs motifs in MEME analysis. Fig. S2. The multiple alignments of deduced amino acid sequences of HMGRs. Fig. S3. Aroma components of 10 varieties in berry skin and berry flesh. Fig. S4. Protein structure and function analysis of VvHMGRs. Fig. S5. Synthetic pathways of main terpenoids in grapes. [file 12870_2021_3073_MOESM1_ESM.docx]

# Expressional diversity of grapevine 3-Hydroxy-3-methylglutaryl-CoA reductase (*VvHMGR*) in different grapes genotypes

**Ting Zheng^1^, Lubin Guan^1^, Kun Yu^2^, Muhammad Salman Haider ^1^, Maazullah Nasim^1^, Zhongjie Liu^1^, Teng Li^1^, Kekun Zhang^3^, Songtao Jiu^4^, Haifeng Jia^1^*and Jinggui Fang^1^***

^1^ College of Horticulture, Nanjing Agricultural University, Nanjing City 210095, Jiangsu Province, PR China

^2^ College of Agriculture，Shihezi University，Shihezi City 832003，PR China

^3^ College of Enology, Northwest A&F University, Yangling 712100, PR China

^4^ Department of Plant Science, Shanghai Jiao Tong University, Shanghai City 200030, Shanghai, PR China


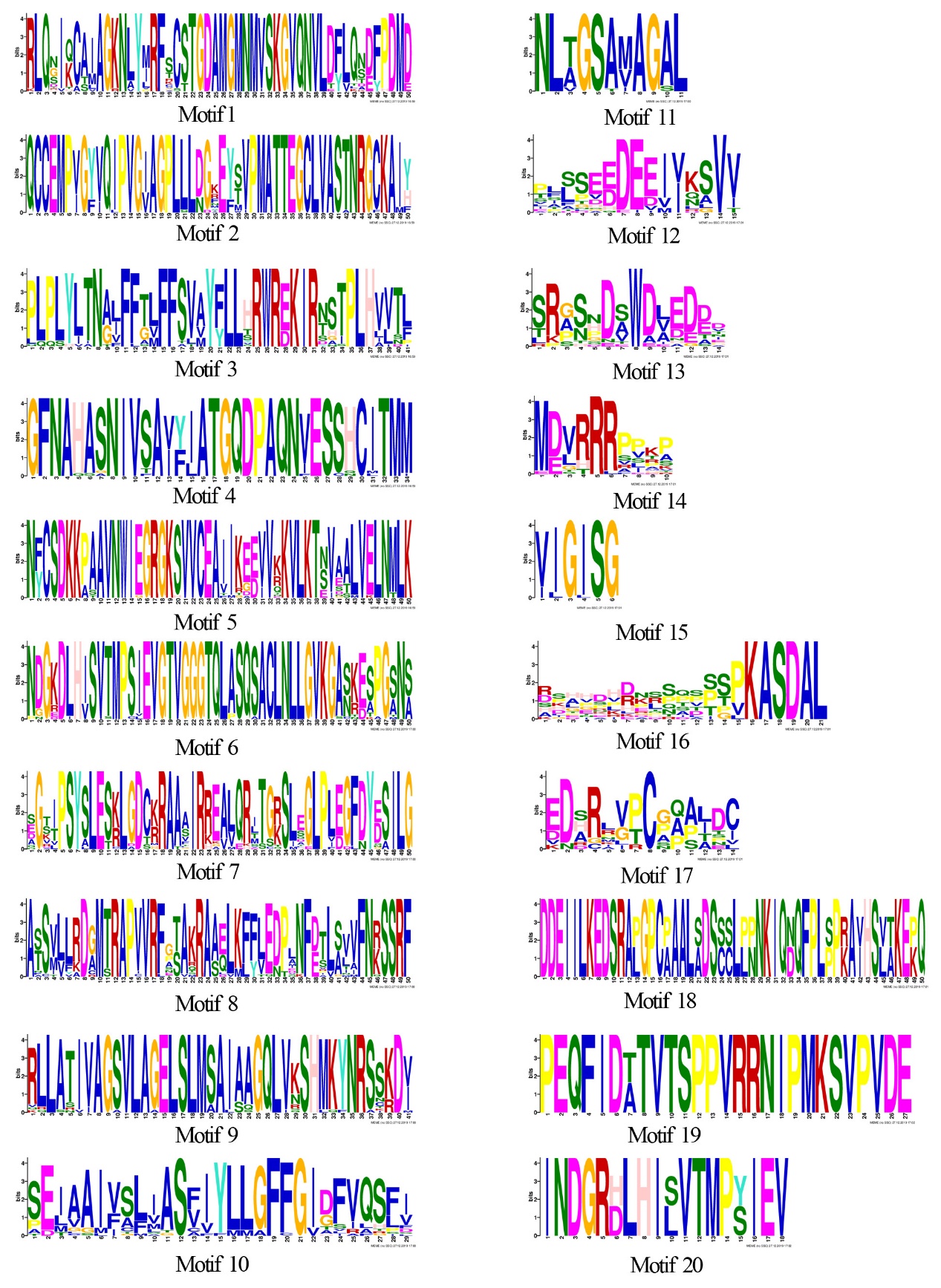


Fig. S1: The amino acid sequence of HMGRs motifs in MEME analysis.


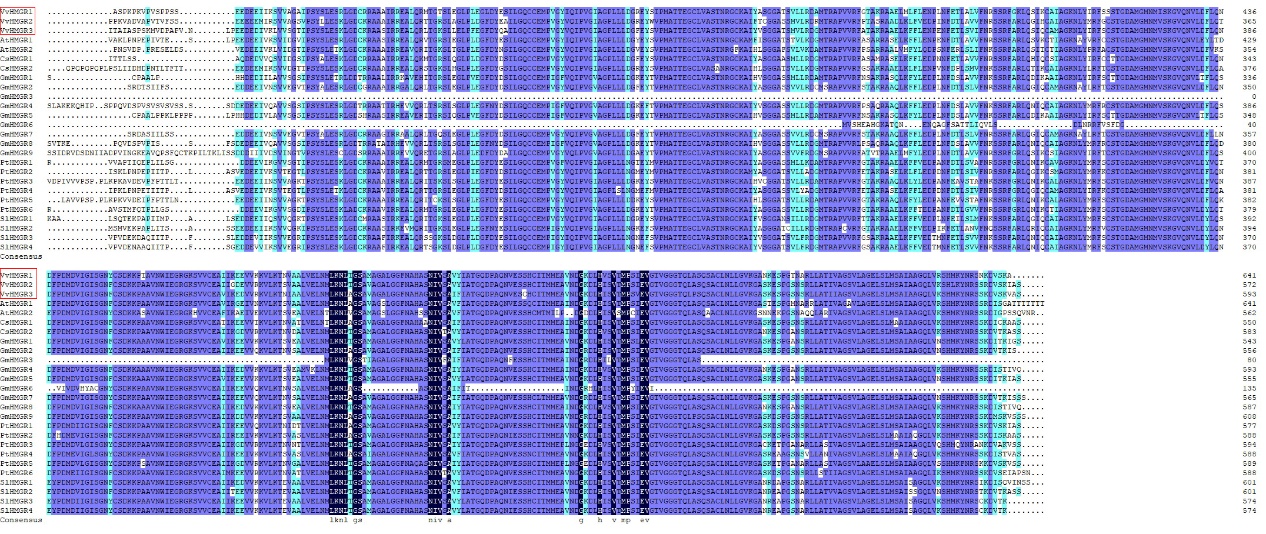


Fig. S2: The multiple alignments of deduced amino acid sequences of HMGRs.


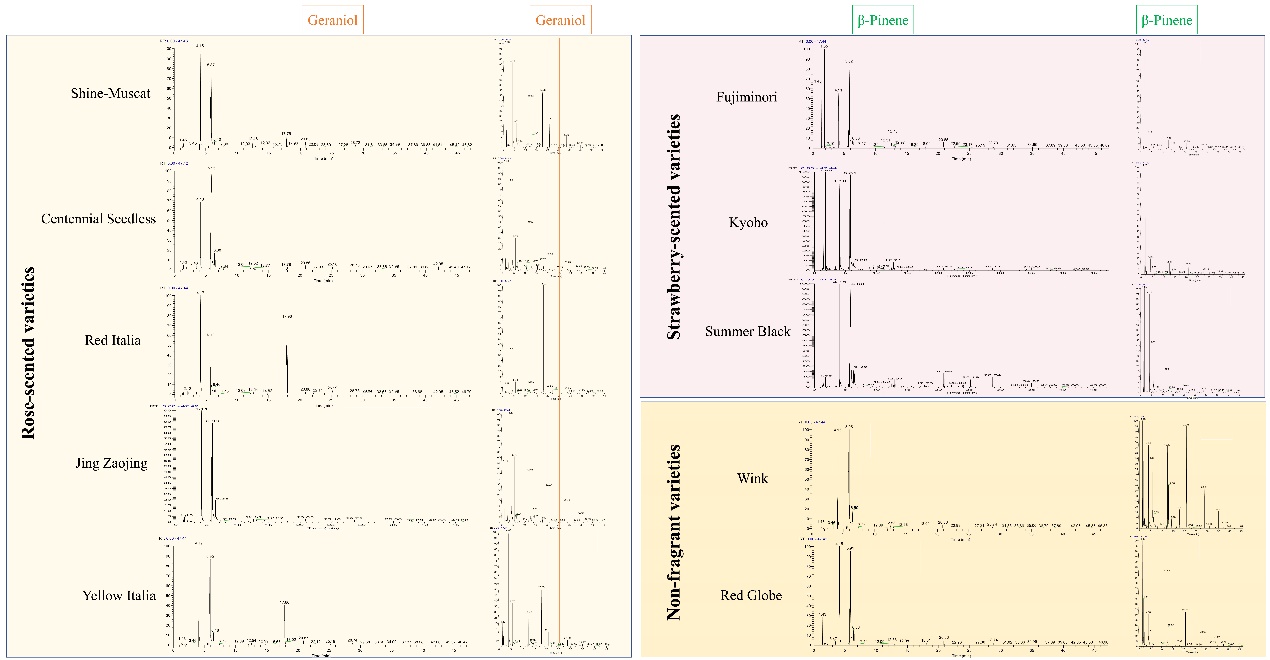


Fig. S3 Aroma components of 10 varieties in berry skin and berry flesh.


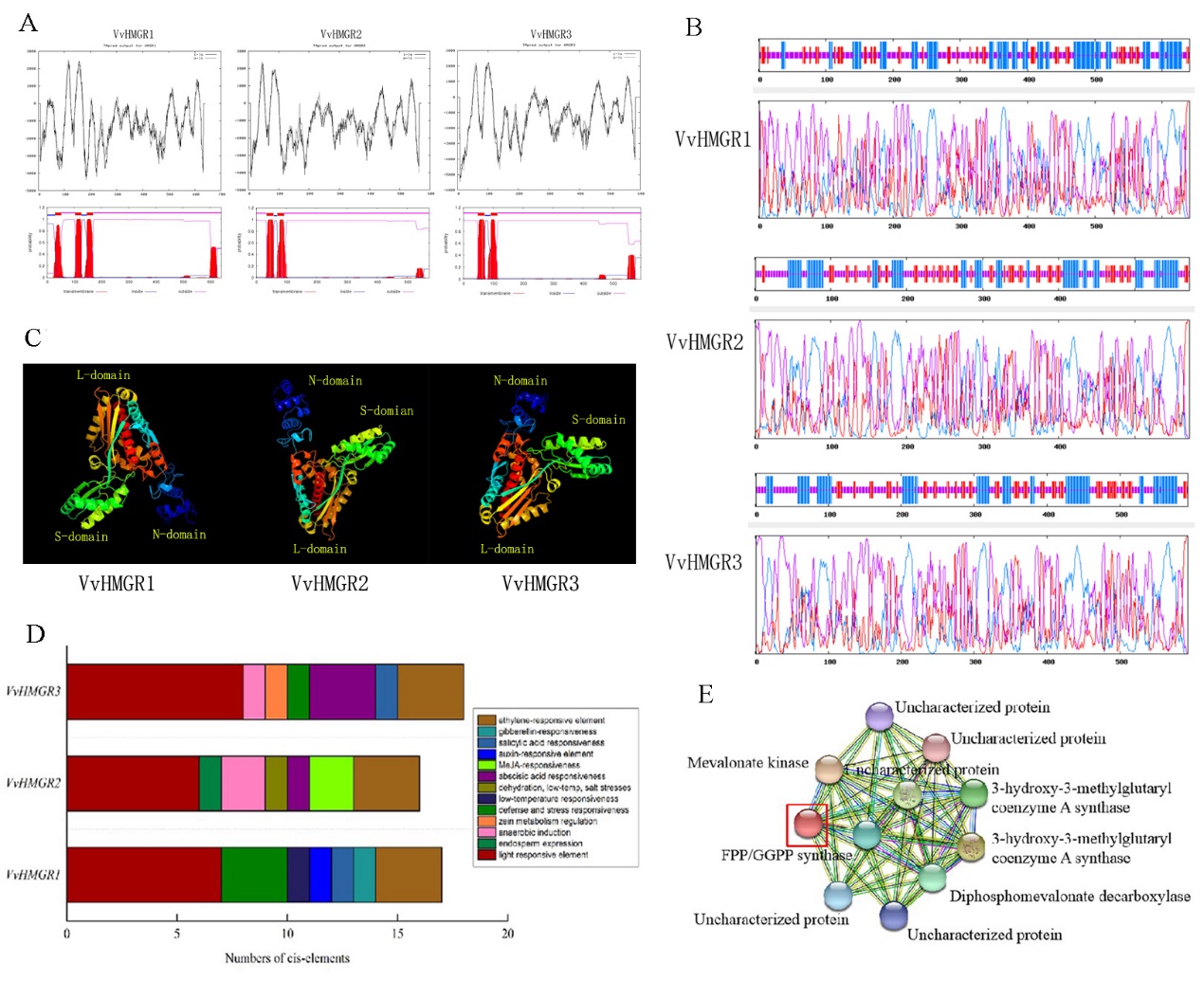


Fig. S4 Protein structure and function analysis of VvHMGRs. A. Transmembrane domain prediction and analysis of VvHMGRs protein; B. Secondary structure prediction of VvHMGRs protein; C. Tertiary structure prediction of VvHMGRs protein; D. Promoter cis-acting element analysis; E. Prediction of interaction protein network.


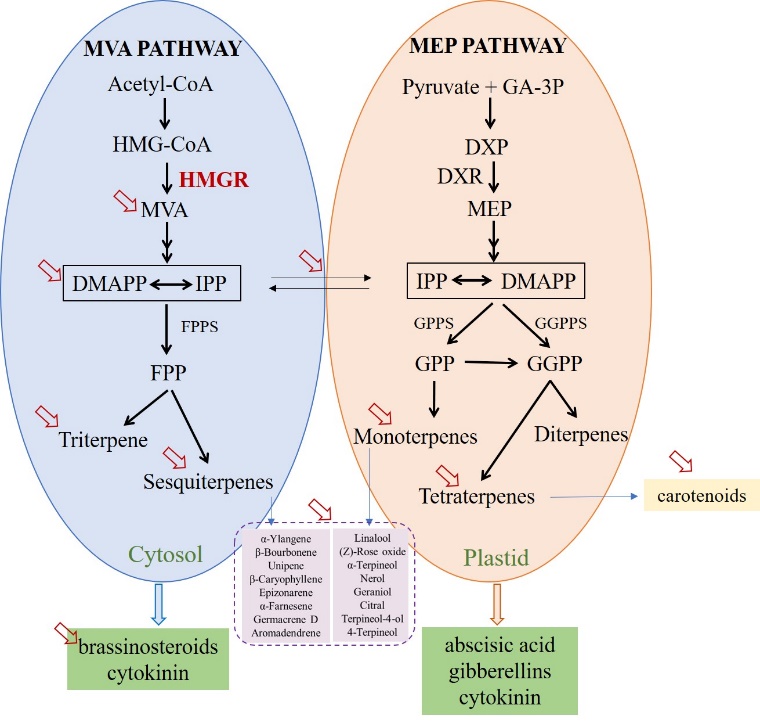


Fig. S5 Synthetic pathways of main terpenoids in grapes. The arrow represents the research results of HMGR affecting the synthesis of terpenoids.
